# Supplementary material for: Assessing the potential of a Bayesian ranking as an alternative to consensus meetings for decision making in research funding: A case study of Marie Skłodowska-Curie actions
Source: PLoS One. 2025 Mar 24;20(3):e0317772. doi: 10.1371/journal.pone.0317772 (PMC11970724; doi:10.1371/journal.pone.0317772)
Supplement: Attachment — Submitted filename: Rebuttal_PlosOne.pdf [file pone.0317772.s001.pdf]

Response to review of

Black Box 'Consensus Meeting': Describing the Differences Between MSCA Consensus Meetings and Bayesian Ranking Recommendations

Rachel Heyard, David Pina, Ivan Buljan, and Ana Marusic

August 2024

Thank you for reviewing our paper. We hereby submit a revised version of our manuscript. Please find below a point by point response to the comments by the three reviewers. We also provide a version of our manuscript highlighting the changes we made. In addition to the requested changes, we have made several wording changes to improve the clarity of the paper.

Reviewer 1

"The title of the paper seems a bit too jargon-laced, which might impact its visibility and downplay its importance. Perhaps a title that better captures the big picture upsides of this analysis, about algorithmic vs. grant panel approaches, and impact on science funding policy would be more fruitful."

Thank you for your comment. We agree and have changed our title to "Assessing the potential of a Bayesian ranking as an alternative to consensus meetings for decision making in research funding: A Case Study of Marie Skłodowska-Curie Actions". First, replying to a comment by reviewer 2, we now write out MSCA. Additionally, the title should now better capture the objective of our study.

This paper provides a cogent contribution to questions about the roles of consensus meetings in grant review, their relationship to individual reviewer reports, lotteries, and considers an alternative more 'algorithmic' approach. This study is interesting and carefully crafted, and I recommend it for publication. I have a number of suggested revisions, all of which are fairly minor. Thank you to the authors for their contribution to science funding policy methodology.

(1) There are a few references missing in the framing of the paper. Some work has been done on consensus meetings and reducing discrepancies caused by gender bias (Bol, T., de Vaan, M. and van de Rijt, A. [2022]: 'Gender-Equal Funding Rates Conceal Unequal Evaluations', Research Policy, 51, available at .), and some work has been done on the inefficiencies of consensus meetings (Erosheva, E., Grant, S., Chen, M., Lindner, M., Nakamura, R. and Lee, C. J. [2020]: 'NIH Peer Review: Criterion Scores Completely Account for Racial Disparities in Overall Impact Scores', Science Advances, 6, available at .) and their implications for lotteries (Lee, C. J., Grant, S. and Erosheva, E. [2020]: 'Alternative Grant Models Might Perpetuate Black–White Funding Gaps', The Lancet, 396, pp. 955–56.).

Thanks a lot for pointing us to those references which we now included in the revised manuscript. We added Bol et al. (2022) in the first paragraph of the *Introduction* section. Erosheva et al. (2020) was added in the *Limitations* section as we thought it fits better after we mention the very specific criteria weighting applied in the MSCA. Lee et al (2020) was added to the *Conclusions* section.

(2) "for some an additional expert had to be consulted to find a consensus." - For clarification, would the additional expert be brought in only in cases with large discrepancies in initial scores?

In very rare cases, an additional expert evaluator was consulted. This would only happen when, due to a very significant difference in individual comments or scores given by the experts, they were not able to agree on a consolidated "consensual" outcome for the proposal being evaluated. For none of the proposals included in our case study, this happened - instead, in our case study, the three experts could always find a consensus.

(3) "to reach a consensus…" - Is the purpose of these meetings to reach a consensus? If so, this seems worth noting (with specific reference to instructions to reviewers, if available/possible). Some grant review panels aren't aiming at consensus explicitly, but rather just giving referees chances to hear their colleagues opinions.

Your comment is referring to an important distinction in the purpose of panel and/or consensus meetings. The meetings for the MSCA are indeed intended to find a consensus. Contrary to reviews of manuscripts submitted to journals for publication, where the editor can send back to the authors reviews with different outcomes, in the MSCA (and H2020/Horizon Europe in general), the final evaluation of a grant proposal consists of a consolidated and consensual set of comments and scores endorsed by the 3 individual experts during the consensus meeting. To make it clear that in our case, the purpose of the discussions was to agree on one consensus score for each proposal, we added the following sentence: "After the consensus meeting each proposal should have a unique consensus score for the three criteria".

(4) "with the most important budget…" - Does this mean the largest budget?

Yes, we mean "largest budget". We changed it in the text to avoid any potential misunderstanding.

(5) It might be interesting to briefly note that there don't seem to be large differences of acceptance/reserve/reject rates despite changes in the amount of reviews required differing by quite a bit.

You are right in your observation. This is due to the way the available funding is distributed across panels for the MSCA. We added the following sentence to explain this: "Since the budget available per panel is chosen to be proportional to the number of proposals submitted to the different panels, these shares are comparable across panels."

(6) "In this setting, the individual evaluation report y_{ij} can be interpreted as the expert's best guess or estimation of the proposal's true quality θ_i" - I understand that this is a controversial point and don't expect the authors to make major revisions to their methodology on this point, but I do think its worth highlighting that this conception of true quality is controversial. For example, see Shaw, J. 2023. "Peer Review, Innovation, and Predicting the Future of Science: The Scope of Lotteries in Science Funding Policy." Philosophy of Science, 90(5): 1297-1306 for the argument that true quality is really a retrospective judgement, rather than expert's best guess, that provides a proposals true quality (although I am adding my interpretation to this paper).

While we agree with your observation, a certain number of assumptions is needed to apply any type of statistical model. We did try to incorporate your reflection by adding this sentence in our *Conclusions*: "Additionally, longitudinal studies relating funding rankings to outcomes of funded projects could help test the underlying assumption that grant peer review effectively estimates the true quality of the proposals, which can only be judged retrospectively [Shaw, 2023]."

(7) "the budget generally determines the number of proposals x that can be funded" - I assume that the budget is fixed prior to the reviewing process, so there's no wiggle room for budget negotiations? If so, this could be made explicit.

Yes, you understood this correctly. We adapted the sentence, to make this clear: "Generally, a predefined budget determines the number of proposals x that can be funded." In our methods section, the explanation of the ranking methodology is kept more general. However, in the MSCA funding decisions, the reserve list represents the only "wiggle room", where an additional set of proposals can be funded if more budget becomes available.
More specifically in the MSCA case, the budget per panel is predefined before the ranking, in a proportional way, based on the number of eligible proposals evaluated in each panel. Then once the ranking is established, the funding is being allocated adding one by one the best ranked proposals until the budget is fully allocated. This means that if we have 10 million euros for a given panel, and the proposals 1, 2, and 3, are budgeted for 3 million each, then the 3 of them will be mainlisted and funded. Whereas, if on the contrary proposals 1, 2, and 3 are each budgeted for 3.5 million, then only the first 2 are funded, whereas the third goes to the reserve list. The reserve list would only be funded at a later stage if additional budget becomes available (for instance due to the failure of the ones ranked above it to conclude in a grant, or if budget from other panels are reallocated by decision of the management board of the funding body). In MSCA, a reasonable number of proposals are kept in the reserve list, as it may happen that several main listed proposals fail to conclude a grant. It usually represents an addition of between 10-20% of the number of mainlisted proposals. For instance if the main list has 50 proposals, then the reserve list will be the 5-10 proposals coming right after, etc.

(8) "Each scientific panel has its own grading behaviour" - Maybe a word or two here adding some specifics would be helpful here.

We added two examples that should give our observation a bit more context. It now reads as such: "Each scientific panel has its own grading behaviour which also varies from call to call, i.e., the Mathematics panel seems to be more reluctant to give high scores in general, and the Social Sciences and Humanities panel gave on average higher IPL scores in 2015 than in the 2019 call. This makes it difficult and even unwise to compare scores of proposals of different disciplines, which were evaluated in distinct panels."

(9) "We would argue that, being such a poor predictor for the consensus report or funding ranking, they are not useful" - It might be worth noting related studies. In the Erosheva et al. study mentioned above, they found that 43% of reviewers changed their preliminary scores as a result of panel discussions.

Thank you for pointing this out. Looking closely at the Erosheva study we read that "Of the assigned reviewers who change their overall impact scores after discussion, only 43% recorded

respective changes in their criterion scores", hence it is not that 43% of reviewers changed their initial scores. Looking into their supplementary material, which they also reference, Table S7 suggests that 47% (20 + 27%) of reviewers change their impact score, regardless of whether they change their criterion score. Hence, this is the number that is relevant for us, and which we included with reference to that Table: "Additionally, a recent analysis of National Institutes of Health funding suggests that, if possible, 47% of reviewers change their initial score after discussion (see Table S7 in Erosheva et al (2020))."

(10) "None of our findings can be generalised to other funding agencies or funding schemes as the analysis focused on a very specific MSCA funding scheme, on two specific call years and tree specific scientific panels" - This might be overstating the case. Perhaps a more modest warning, like saying that generalizations should be cautious to consider the specifics of the MSCA funding scheme, would be appropriate.

We agree, and adapted the paragraph, to make our claim a bit less strong.

Reviewer 2

The manuscript highlights an important and underdeveloped area of funding practices. The authors have adequately reported the uncertainty in this field and suitably reference past evidence in this space. I have some additional comments under each of the manuscript headings, see below.
For those who may not know what the MSCA is I think this needs to be reported in full in the title.
Thank you for reviewing our manuscript.
As suggested by Reviewer 1 we have adapted our title, and at the same time, also now write out MSCA.

Abstract: There is no background in the abstract to put this research into context. Understanding the unmet need and the reason for the research/study would strengthen the abstract.
Thank you for your comment. We followed the PLOS ONE submission guidelines, which do not ask for a background in the abstract. We understand however that this can be very useful for our readers, and two sentences introducing the need for our study. Given the imposed word count limit of 300 words, this is all we could add.

Introduction: Providing some context around describing the allocation of funding as simplistic and ignoring the underlying uncertainty needs elaboration. It is not clear what this is referring to and given the existing evidence in this space, I would suggest updating the 2008 reference used. The authors adequately provide context around alternative approaches and how this current work fits into the wider context. Perhaps, providing the three research questions to the introduction (or methods) would make it easier to see the purpose of the analysis.
Thank you for this - actually the context for this first sentence comes in the following sentences. Therefore, we added a "Specifically," to make this clear. We also added a sentence on the documented low inter-rater reliability in grant review, to further explain why averages that ignore the uncertainty are ill-advised. We left the 2008 paper in-there since we could not find a better, more recent reference. Following your suggestion, we added the research questions at

the end of the introduction and updated the “Research questions and data analysis” section to avoid any duplicates.

Methods: the authors provide a thorough account of the approach taken. Who conducted the analysis and the software used for analysis is not reported though, which would help others to replicate in the future. Perhaps, move the research questions to the beginning of the methods section so the reader is able to see this first. Although there is a data analysis subheading there is minimal information provided, albeit a link to OSF protocol. Listing the questions outlined in the protocol would be very useful in the manuscript (at the end of the introduction or as part of the methods). Having them earlier in the manuscript would then support the information detailed in Box 1.
As per your suggestion in the previous comment we moved the research questions to the end of the introduction. The analysis was conducted by the first author (Rachel Heyard), as can be seen in the *Author contributions* section. Thank you for your remark on the fact that the software usage was not clear. We already had a *Data availability* statement in our first submission mentioning that we used R, and that the code is available on gitlab.com. We did however not mention the main R-package (ERforResearch) that was used and needs an installation of JAGS. We updated the *Data availability* statement to a *Software and data availability* statement instead and added this information.

Why was the data from 2015-2019? As this is now 5 years ago and have processes/practices changed during this period. Explaining why the analysis was with 5 year old data needs to be reported within the manuscript to be able to place the analysis in the wider context.
At the initiation of this project, which was at the end of 2022, only limited data was available from the new Framework Programme (Horizon Europe). Due to the differences in the scope and processes of Horizon Europe and Horizon 2020, the framework programme we used here, we decided not to merge the data together, and since, by that time only one evaluation year was completed and available from Horizon Europe we decided to use only Horizon 2020 in our analysis. We added a sentence on this in the section *Data source: The MSCA European Training Networks*: “Note that after 2020, ….”. A follow-up analysis on Horizon Europe data could be informative regarding the generalizability of our findings to other European funding calls, and we added this sentence in the *Limitations*: A follow-up analysis on the newer Framework Programme, Horizon Europe, could help further investigate the generalisability of the findings.

The three research questions are adequately reported in the results section, along with tables and figures to further explain the results of the analysis.
Thank you for your comment. We also mention the research questions earlier at the end of the introduction.

The discussion relates back to the three research questions, although I'm unsure of the second paragraph and the justifications around the usefulness of individual evaluation reports. Further explanation is required, to back up the statement based on the results of the analysis. It is also challenging to understand what this paragraph is trying to say, and its relation to existing evidence. The authors provide limitations of the analysis, although the last three sentences

appear out of place. It would be better placed as a final paragraph to summarise the analysis in the wider context of funding allocation, how funders can use this in future considerations (along with caveats) and what does it mean in terms of bias. As noted, points raised in the introduction around the challenges with funding allocation (inefficient, biased, 'black box') need to be picked up in the discussion. In particular, the 'black box' terminology, mentioned in the introduction is not mentioned throughout the manuscript.
We revised the second paragraph of the discussion and hope it is a bit clearer now. Regarding the last sentences in the *Limitations*, we agree and moved the last two sentences on “further research” to the *Conclusions*. The third last sentence is, in our opinion, a limitation of statistical models in general when dealing with skewed data.

Using algorithms to attempt to remove bias and inefficiencies also has limitations, which is mentioned in the existing literature. Balancing funding allocation approaches is challenging, yet can complement each other given the right context. I feel the authors could pick up these opportunities in the manuscript more and tense out how and in what circumstances it would be acceptable to use algorithms to inform funding allocation processes and ensure these practices are transparent, fair and of high quality. How this analysis aligns and offers these opportunities more widely could be strengthened in the manuscript.
We agree. We emphasise in our *Discussion* that we could see the commission use such an algorithmic approach for a triage of the worst ranked proposals before the consensus meetings. In our revised *Conclusions* we then further put the algorithmic approach in the context of bias and unreliability. We argue that further research and experimentation, specifically from funding agencies, is needed to ensure no social disparities and biases are introduced in the process.

Minor corrections: There are typo errors on the figures such as 1,006 instead of 1'006 and 7,870 instead of 7'870
Typo error - [Pina et al., 2015, Graves et al. [2011], Erosheva et al. [2021]].
Thank you for such a careful review of our manuscript. We addressed those errors and revised the manuscript accordingly.

Reviewer 3

Technical Soundness:
The manuscript is partly technically sound. The methodology, involving a Bayesian hierarchical model to compare individual evaluation reports (IER) and consensus meeting reports (CR), is robust and well-chosen for the research questions posed. The study design includes appropriate controls and comparisons, with an adequate sample size across multiple panels and call years. However, while the Bayesian model is rigorously applied, the generalizability of the findings to other funding schemes and contexts remains uncertain. Detailed case studies and further validation of the model using additional datasets could enhance the robustness of the conclusions.
Thank you for your comment. We agree with your interpretation. Our limitation section addresses the uncertain generalisability. And we call on more funding agencies to perform additional research to further contextualise our findings.

Statistical Analysis:
The statistical analysis has been performed appropriately and rigorously. The use of a Bayesian hierarchical model to account for variability and uncertainty in the data is commendable. The manuscript provides a clear explanation of the statistical methods used, and the comparative analysis between the Bayesian ranking and consensus meetings is thorough and well-supported by the data.
Thank you.

Data Availability:
The authors have made all data underlying the findings in their manuscript fully available. This transparency is crucial for the reproducibility of the research. The data is openly accessible through the provided links, ensuring that other researchers can verify and build upon the study's findings.
Thank you.

Manuscript Presentation:
The manuscript is presented in an intelligible fashion and written in standard English. The structure is logical, and the writing is clear, making the complex statistical methodologies and findings accessible to a broad audience. However, the manuscript could benefit from a more detailed discussion on the potential biases in both the Bayesian model and consensus meetings.
We did not include a discussion or analysis on potential biases in our manuscript for two reasons: (1) we do not have the data to perform an analysis, and hence any discussion would be based on speculations, and (2) we want to keep the focus of our analysis on the comparison between the scores, ranking and funding decisions retrieved from the Bayesian ranking methodology and the consensus meetings. However, it is important to note that both the Bayesian ranking and consensus meetings, are somehow depending on evaluation scores, which are inherently subjective and therefore likely to be biased. We added a sentence in the *Discussions* on the fact that the Bayesian ranking was initially suggested to decrease the subjectivity in the decision making process.

Additional Comments:
Generalizability: The study focuses on the MSCA funding scheme and a limited number of panels. A discussion on how the findings could be generalized to other funding mechanisms would be beneficial.
We commented on the generalisability issue in our limitation section and call for more research on this topic.

Bias Analysis: A thorough analysis of potential biases in expert evaluations and consensus discussions could provide valuable insights and strengthen the manuscript's conclusions.
While interesting, we believe that this is out of scope for our manuscript. On top of that, the data we used for our analysis, as agreed upon with the Commission, does not include much additional information on demographics of the applicants or the reviewers. We did however, in the process of this revision, discuss biases in the grant peer review process and that funding agencies should be cautious not to create biased systems: "[...] *especially since differences in the*

process of weighting criteria scores into a total score could potentially penalise certain applicant groups [Erosheva et al. (2020)]" - in the *Limitations* - and "[...] *while ensuring to not introduce any social disparities [Lee et al. (2020)]* - in the *Conclusions*.

Future Research: Outlining specific future research directions to test the applicability of the findings in other contexts and with different types of funding mechanisms would be a valuable addition.
Thank you for the suggestion. We extended the paragraph on further research in the revised manuscript.

While the manuscript is technically sound and well-written, addressing a few areas could enhance its clarity and robustness. Specific improvements include a more detailed discussion on the generalizability of findings, potential biases in both the Bayesian model and consensus meetings, and future research directions. Addressing these points would make the manuscript even stronger.
Thank you for your helpful comments. We were able to address them, as outlined in our replies above.
