# Supplementary material for: Assessing the potential of a Bayesian ranking as an alternative to consensus meetings for decision making in research funding: A case study of Marie Skłodowska-Curie actions
Source: PLoS One. 2025 Mar 24;20(3):e0317772. doi: 10.1371/journal.pone.0317772 (PMC11970724; doi:10.1371/journal.pone.0317772)
Supplement: Attachment — Submitted filename: Rebuttal_PlosOne_November.pdf [file pone.0317772.s002.pdf]

Response to review of

Assessing the potential of a Bayesian ranking as an alternative to consensus meetings for decision making in research funding: A Case Study of Marie Skłodowska-Curie Actions

Rachel Heyard, David Pina, Ivan Buljan, and Ana Marusic

November 2024

Thank you for reviewing our paper. We hereby submit a revised version of our manuscript. Please find below a point by point response to the comments by the reviewer. We also provide a version of our manuscript highlighting the changes we made.

Reviewer

In the introduction section, the author is requested to discuss the theoretical and methodological foundations of existing research, the gaps in existing research, and the areas in which this article will advance existing theories and methods.

Thank you for your comment. We believe our introduction discussed existing research and the motivations for our study. The gaps in the existing research are now emphasised with the sentence we added at the end of the second paragraph. The specific areas that our study will advance are explained at the end of the third paragraph:

We specifically use the BR to determine the contexts in which such a substitution would be advantageous and the scenarios in which it may be less suitable. This study provides valuable insights into whether the outcomes or decisions derived from the consensus meeting can be predicted from the individual evaluation scores alone, or whether the dynamics of the consensus meetings are unpredictable solely from the individual evaluation scores. Our results contribute to enhancing our knowledge of the feasibility and effectiveness of such algorithmic approaches in grant proposal evaluations, ultimately facilitating evidence-based decision-making in research funding allocation and making the process more transparent and efficient for expert evaluators, panel members and applicants.

In the conclusion section, please have the author discuss the theoretical and methodological contributions of this article

We believe that the contributions of our article are described in the *Discussion*, but we now added an opening sentence to the *Conclusions* that further emphasises the contribution: *Our study offers novel insights into the dynamics of consensus meetings and the potential of algorithmic approaches to effectively aggregate individual expert evaluations as an alternative to traditional consensus meetings.* We also did minor changes to the *Conclusions* section to improve the flow.
